# On the Climate of the Neilgherry, or Blue Mountains of Coimbatoor, South India

**Published:** 1830-10-01

**Authors:** 


### CLIMATE OF THE NEILGHERRY, OR BLUE MOUNTAINS OF COIMBATOOR, SOUTH INDIA. By *James Hough*, of Madras. Octavo, pp. 172. Hatchard, 1830.

THE baleful influence of an East-India climate on the constitutions of our countrymen and women is now too well known. This it is which often compels many valuable public officers prematurely to retire from a service, in which all their temporal hopes were centered—and that, perhaps, at the very time when their opening prospects were beginning to reward their toils and their assiduity. To men rendered incapable of performing their duties, suffering from disease of body and depression of mind, it must be no trifling gratification to know that, within the territories of British India, there exists a region singularly salubrious, romantic, and beautiful, where they may, with safety and with little difficulty, retire to recruit their health and vigour, without the misery and expense of time and money attendant on a long sea-voyage to Europe.

The Neilgherries were scarcely known before the year 1819, when several gentlemen, then residing at Coimbatoor, explored them, and published some accounts of these singular mountains in the India newspapers. These mountains are situated to the north-west of Coimbatoor, about eleven degrees from the Equator, and ranging 40 miles in length by 15 or 20 in breadth—the highest part being about 8000 feet above the level of the sea. The following extract from the letter of a medical gentleman, who examined the topography of this region, will be read with interest.

"The salubrity of this climate has now been fully ascertained. The incredulity that for some time prevailed on the subject was noticed in my first communication; and, considering the general prevalence of fever in mountainous regions throughout India, it must be conceded that a degree of scepticism was, for a time, not unreasonable. But it is quite inexcusable to remain incredulous after the numerous favourable reports that have been made by eminent medical officers. However, I will endeavour to enable your readers to draw the same conclusion; for this inference may be anticipated from every reasonable man, that if my description of the country be correct, *it must be the region of health.*

In the first place, it is entirely free from those morasses and vast collections of decayed vegetables that generate miasma, which, some are of opinion, is the principal cause of fever in other mountainous regions of India. This cannot be said indeed of the *passes* to the Neilgherries throughout the year; since, from February to May inclusive, their climate is as insalubrious as that of other parts of the Ghauts. But no inconvenience is to be apprehended from passing through them even at that season, unless the traveller be so imprudent as to halt there during the night and sleep; and as this may always be avoided by starting at a proper hour with sufficient means of conveyance, the existence of fever in the passes forms no exception to the favourable character given of the climate at the summit. During my residence there, two cases of the kind occurred, both which appear to have been occasioned by zeal for the public service, which detained the gentlemen longer in the unwholesome parts, than was, perhaps, absolutely necessary. However, on re-ascending the hills, both recovered." 28.

The fatal cholera has never ascended these mountains. In equability of temperature, this place surpasses that of any other country with which we are acquainted. The average range of the thermometer is about thirty degrees below that of the adjacent coasts of Malabar or Coromandel.

"In 1825, the frost commenced on the 11th of September, and prevailed, with some intermissions, until the end of the following March. This was unusually early, as it does not often appear before the middle of October. The sight of the ground covered with hoar frost is highly gratifying to an Englishman, as it revives pleasing associations that had, probably, not recurred to his mind since he left his dear native land; and, if I may be allowed to judge of others by myself, he is not displeased to have his fingers pinched by the frost, at least until this sensation ceases to be a novelty. The pools and sides of streams in the valleys are frequently covered with a thin coat of ice, which for some time resists the influence of the sun's rays. Standing water is generally frozen, and on the 13th of February I found some ice an inch and a half thick." 37.

The scenery of this interesting country it is difficult to describe in adequate language.

"It presents very little of that bleak, rugged, and barren appearance which is common to most other mountainous regions. Peringa and Maika Naads, are composed of mountains which vary greatly in their elevation. Some of these eminences are almost perpendicular, towering to the clouds, and descending in deep and terrific precipices. Their sides are occasionally bare, but more frequently covered with fine grass, a rich profusion of plants, and a short brushwood, with almost every variety of fern.

Numerous streams are seen meandering through the valleys, and might easily be turned in all directions to irrigate the fields that skirt their margins; but the inhabitants avail themselves very little of this advantage. The bold eminences are surrounded by smaller hills, whose gentle declivities are adorned with patches of cultivation, and whose ravines are filled, and summits often crowned, with groves of majestic trees." 20.

We shall conclude this short notice of the "Blue Mountains" of Hindostan with the following extract.

"It appears by the registers which accompanied my third letter, that the *maximum* heat in the shade, at noon, during fourteen months, was 68° Fahrenheit. The thermometer rarely stood so high. The average for the year has been ascertained to be about 56¾°, whilst the extreme variation is only 12°: that is from July to March the thermometer will occasionally stand at noon, both in and out of doors, at 56°; during the remaining three months it will rise as high as 68°. There are exceptions to this estimate; the general rule is, that the thermometer does not vary two degrees, very often not a line, in a well-sheltered house, during the twenty-four hours. A visitor was once so struck with this fact, that he seriously asked whether any thing was the matter with a thermometer which hung in his room, as he had never observed the mercury to move during his residence here. The temperature before sunrise is seldom above 50°. For many months in the year it sinks to the freezing point, occasionally below it. It was marked at 19¾ for three successive mornings of December 1825. Occasionally frosts are seen as early as September, and in every succeeding month until April, in the absence of rain. At times the sun is felt to be extremely oppressive in situations sheltered from the wind; but the thermometer rarely indicates a heat above 76°. The interposition of a cloud or a passing breeze, reduces the temperature considerably. Exercise may be taken on horseback by persons in health, at all hours of the day.

During the prevalence of both monsoons, that is, from the middle of June till the middle of December, very boisterous and unpleasant weather is occasionally experienced. It seldom, however, is so bad as to prevent a person from going abroad during some portion of the day. The nights are uniformly cold, so that blankets for the bed can never be dispensed with, and fires in the morning and evening are comfortable during a great part of the year, and occasionally throughout the day. These are the results of seven years' experience of the climate of the Neilgherries. In what other part of the globe is such a climate to be found? Certainly not at the Cape, where the benefit derived by invalids in the cold season is very generally lost in the hot. The same remark applies to most parts of the Isle of France. In the south of Europe, in France, even in our own country, summer heats are occasionally excessive, and the variations of climate throughout the year very great. The temperature of Van Dieman's land appears to approach nearer to that of the Neilgherries than any other we are acquainted with; but an attentive comparison of their thermometrical registers kept there will show, that even that highly favoured region does not possess a climate at once so equal and so cool throughout the year as that of these mountains.

We have then, within our own territories, a region blessed with an unequalled climate, highly beautiful and fertile, and of easy access from all the Indian Presidencies. By a road which is now in progress, the distance from Ponnany to the most elevated part of the Neilgherries that is inhabited will be about eighty-five miles. Ponnany is a sea-port on the western coast, about thirty miles south of Calicut. An invalid, therefore, leaving Calcutta at any time after the rains, would have a fair-weather voyage of fifteen or twenty days, at an expence of two hundred rupees, to the coast; and from thence, four very easy night-journeys in a palankeen would carry him to the summit of the hills. From Bombay the sea-voyage at the same season would be made in less than half the time. During the prevalence of the south west monsoon, the most eligible place of landing from both Presidencies would be Negapatam, on the eastern coast, which is eighty miles distant from Trichinopoly; and the traveller would be conducted along two hundred miles of good road, and through a populous country to the hills." 132.

There is reason to believe that the Neilgherry Mountains will prove of more importance to Britons than may, at first sight, appear. There are many people in India who have no wish to revisit their native soil—still more who dread its climate, after a long residence between the Tropics—and others still, who have not the means to return home for health. To all these, the Neilgherries present a desirable retreat in which to spend the remainder of their days, or recruit those powers of the animal machine, and even of the drooping spirit, which have been prostrated beneath the burning suns of our Indian possessions. The easy access to such an "ASYLUM OF HEALTH," too, must prove a great source of consolation and hope to those who are embarking for the shores of Hindostan.

